# MSH2 ATPase Domain Mutation Affects CTG•CAG Repeat Instability in Transgenic Mice

**DOI:** 10.1371/journal.pgen.1000482

**Published:** 2009-05-15

**Authors:** Stéphanie Tomé, Ian Holt, Winfried Edelmann, Glenn E. Morris, Arnold Munnich, Christopher E. Pearson, Geneviève Gourdon

**Affiliations:** 1 INSERM, U781, Université Paris Descartes, Hôpital Necker-Enfants Malades, Paris, France; 2 Wolfson Centre for Inherited Neuromuscular Disease, RJAH Orthopaedic Hospital, Oswestry, Shropshire, United Kingdom; 3 Institute of Science and Technology in Medicine, Keele University, Keele, Staffordshire, United Kingdom; 4 Department of Cell Biology, Albert Einstein College of Medicine, Bronx, New York, New York, United States of America; 5 Program of Genetics and Genome Biology, The Hospital for Sick Children, Department of Molecular Genetics, University of Toronto, Toronto, Canada; University of Minnesota, United States of America

## Abstract

Myotonic dystrophy type 1 (DM1) is associated with one of the most highly unstable CTG•CAG repeat expansions. The formation of further repeat expansions in transgenic mice carrying expanded CTG•CAG tracts requires the mismatch repair (MMR) proteins MSH2 and MSH3, forming the MutSβ complex. It has been proposed that binding of MutSβ to CAG hairpins blocks its ATPase activity compromising hairpin repair, thereby causing expansions. This would suggest that binding, but not ATP hydrolysis, by MutSβ is critical for trinucleotide expansions. However, it is unknown if the MSH2 ATPase activity is dispensible for instability. To get insight into the mechanism by which MSH2 generates trinucleotide expansions, we crossed DM1 transgenic mice carrying a highly unstable >(CTG)_300_ repeat tract with mice carrying the G674A mutation in the MSH2 ATPase domain. This mutation impairs MSH2 ATPase activity and ablates base–base MMR, but does not affect the ability of MSH2 (associated with MSH6) to bind DNA mismatches. We found that the ATPase domain mutation of MSH2 strongly affects the formation of CTG expansions and leads instead to transmitted contractions, similar to a *Msh2*-null or *Msh3*-null deficiency. While a decrease in MSH2 protein level was observed in tissues from *Msh2^G674^* mice, the dramatic reduction of expansions suggests that the expansion-biased trinucleotide repeat instability requires a functional MSH2 ATPase domain and probably a functional MMR system.

## Author Summary

Myotonic dystrophy type 1 is a neuromuscular disease characterized by highly variable clinical manifestations, including muscular and neuropsychological symptoms. DM1 results from the dramatic expansion of an unstable CTG repeat in the *DMPK* gene. Longer CTG repeats cause a more severe form of the disease and an earlier age of onset. The DNA mismatch repair proteins MSH2 and MSH3 are known to be major players in the formation of trinucleotide expansions. Nevertheless, the mode of action of these proteins remains elusive. In order to get further insight into the role of MSH2 in the formation of CTG expansions, we used a mouse model carrying a mutation in the conserved ATPase domain of *Msh2*. This mutation affects the function of this domain and alters the DNA repair mismatch activity. After breeding of these mice with mice carrying highly unstable CTG repeats, we found that the ATPase domain mutation of MSH2 strongly affects the formation of CTG expansions. Our findings show that expansion-biased trinucleotide repeat instability requires a functional MSH2 ATPase domain and support the hypothesis, according to which a functional MMR activity is required to generate expansions.

## Introduction

Myotonic dystrophy type 1 (DM1) is a neuromuscular disease characterized by highly variable clinical manifestations including skeletal muscle, cardiorespiratory and brain defects and endocrine abnormalities [1]. DM1 results from the expansion of an unstable CTG trinucleotide repeat in the 3′-untranslated region of the dystrophia myotonica protein kinase (*DMPK*) gene [2]–[4]. In normal individuals, the CTG repeat at the DM1 locus varies from 5 to 37 repeats and remains stable over generations. Tract lengths of >50 CTG repeats are dramatically unstable in successive generations, with a strong bias towards expansions, often reaching thousands of CTGs in the most severe form of the disease [1]. The level of CTG repeat instability depends on the sex and repeat length of the transmitting parent [5],[6]. The size of the expanded repeat inversely correlates with the age at onset and is positively correlated with the severity of symptoms providing the molecular basis for anticipation [7]. In addition to intergenerational instability, the CTG repeat is also somatically unstable in DM1 patients. Somatic instability, which is age-dependent, biased towards expansions of CTG repeat length, presents variable expansion patterns between tissues, and is probably associated with the progression of disease symptoms in DM1 patients [8]. A better understanding of the molecular mechanisms underlying trinucleotide repeat instability is fundamental for the development of therapies aimed at controlling trinucleotide repeat instability. Such therapeutic approaches would hopefully reduce the disease severity and progression of the symptoms.

Extensive analysis of repeat instability in various model systems *in vitro* and *in vivo* has revealed crucial insights into the role of *cis*-elements, DNA replication and genome-maintenance repair [9]–[12]. Numerous transgenic mouse models and their derived cell lines revealed that the repeat expansion rate does not always correlate with cell division rate, suggesting the involvement of various replication-independent mechanisms [13],[14]. Studies have reported that several mismatch repair (MMR) proteins are involved in expansion-biased trinucleotide instability in transgenic mouse models carrying expanded CTG•CAG repeat tracts. Accumulation of CTG•CAG expansions in mitotic, non-mitotic and germline tissues clearly depends on the MMR proteins MSH2, MSH3 and to a lesser extent on PMS2 (postmeiotic segregation homologue 2) [15]–[20]. Precisely how MutS homologues MSH2 and MSH3 mediate CTG•CAG repeat expansions has not yet been clearly determined.

MMR proteins are required to maintain genomic integrity in prokaryotes and eukaryotes, by correcting single mismatches and short unpaired regions, such as small insertions and deletions [21],[22]. In eukaryotes, three proteins are involved in mismatch recognition, MSH2, MSH3 and MSH6. The three proteins form two heterodimers MutSα (MSH2-MSH6) and MutSβ (MSH2-MSH3). MutSα is thought to be involved primarily in the recognition and repair of base-base mismatches and small insertion/deletion loops. MutSβ acts preferentially on insertion/deletion loops up to 12 nucleotides in length [23]. MMR is tightly linked with the mismatch-binding-dependent ATPase activities of the MSH complexes but the precise roles of these activities are still in debate. Upon binding to the mismatch, the MSH complex exchanges a bound ADP for an ATP, which permits a change in the proteins conformation and the signaling of downstream repair events [21],[22],[24],[25]. The MSH2, MSH3, and PMS2 mismatch repair proteins are also involved in other DNA repair pathways such as single-strand annealing and homologous recombination, anti-recombination, DNA damage signaling, apoptosis, as well as site-specific mutagenesis during immunoglobin somatic hypermutation and class switch recombination [26]–[29]. While the MSH2 ATPase activity is known to be required for some of these non-MMR functions, the specific roles of ATP-binding and ATP-hydrolysis remain to be elucidated.

Different scenarios have been suggested to explain the role of MMR proteins in trinucleotide repeat instability. It has been shown that CTG•CAG repeat tracts can form hairpin structures and/or slipped-strand structures *in vitro* [30],[31]. These structures are suspected to disturb DNA metabolism *in vivo*, leading to trinucleotide repeat instability [9]. An initial study reported that *in vitro* MSH2 can recognize and bind to slipped-strand (S-DNA) structures formed by expanded CTG•CAG repeat tracts [32]. More recently, a detailed study of MutSβ binding *in vitro* to long CAG hairpin structures was reported [18], as well as an *in vitro* assay to process slipped-DNAs [33].

Three models explaining the mechanism through which the MMR proteins MSH2 and MSH3 act to generate CTG•CAG expansions have been put forth. The first model proposes that the role of the MMR proteins MSH2-MSH3 (MutSβ) in trinucleotide repeat expansions is through protecting CAG loops from repair by binding the loops [18]. This model was based upon the reduced ability of the human MutSβ complex to hydrolyze ATP when bound to hairpins formed by either (CAG)_13_ or (CTG)_13_; suggesting that the protein complex would be stuck on the loop, unable to translocate along the helix, and unable to signal or interact with downstream repair proteins. In this manner binding of MutSβ to CAG or CTG hairpins would abort their repair, thereby allowing the loops to be incorporated into the genome as expansions. Thus, in this model MutSβ binding but not ATP hydrolysis would be critical for expansions.

The second model posits that the MutSβ complex is not required for CTG or CAG slip-out processing or protecting them from repair, but rather MutSβ may be involved in expansion prior to the processing of the slip-outs [33]. An *in vitro* repair assay using slipped-DNAs with slip-outs of either (CAG)_20_ or (CTG)_20_ revealed that these can be correctly repaired, escape repair, or be repaired in an error-prone manner (only partially excising the slip-outs). All of these processes occurred in human cell extracts deficient in either MSH2 or MSH3. These authors suggested that rather than acting in the processing or protecting CTG or CAG slip-outs from repair, MutSβ may be involved in forming the slip-outs. It is unknown if such an activity would require the endogenous ATPase activity of the protein.

The third model suggests that the mismatch repair process itself is involved in trinucleotide repeat expansions [17]. This study demonstrated that the MutL MMR homologue PMS2 is also involved in CTG•CAG repeat somatic expansions in mice. Some repeat expansions are still formed in PMS2-deficient mice suggesting a partial role of PMS2 in trinucleotide repeat instability [17]. The authors proposed that the MMR activity is required to generate somatic expansions implying that the role of MMR proteins would not be limited to the binding of MutSβ complex to the A-A mismatches in CAG hairpins and/or to the MutSβ-mediated formation of slipped-strand structures. In this model the ATPase activity of the MutSβ would be critical to expansions.

In an attempt to distinguish between the different hypotheses and to clarify the role of the ATPase activity of MSH2 in the mechanism of CTG•CAG expansion, we crossed *Msh2* ATPase-defective mice (*Msh2^G674A^* mice) with mice carrying a large unstable CTG expansion (DM300-328) [27],[34],[35]. The DM300-328 mice carrying >300 CTG repeats in the context of the human genomic *DM1* locus of over 45 kb display very high levels of intergenerational and somatic instability similar to that observed in DM1 patients [36]. The *Msh2*-mutant mice carry a missense mutation of a glycine-to-alanine within the Walker “type A” motif GXXXXGKS/T of the MSH2 ATPase domain [27],[34] . This ATPase domain in MSH2 and in many proteins with ATPase function, is known to coordinate the phosphate groups of ATP [34]. Mismatch binding experiments using *Msh2^G674A/G674A^* ES cell extracts demonstrated that the mutant MSH2^G674A^-MSH6 complex retained normal mismatch binding activity. However, the complex was resistant to ATP-mediated mismatch release and had lost its capacity to signal mismatch repair resulting in MMR-deficiency similar to that observed in *Msh2^−/−^* null mutant extracts. Moreover, the homozygous ATPase mutation caused a mutator phenotype, increased genome-wide mononucleotide and dinucleotide instability in the genome of mutant mice similar to that observed in *Msh2* ^−/−^ mice. However, in contrast to the *Msh2*-null allele, the *Msh2^G674A^* mutation did not significantly affect the cellular response to DNA damage-inducing agents, indicating that normal ATP processing with subsequent repair is not essential for the apoptosis signalling function of MSH2 [34]. Therefore, *Msh2^G674A^* mutant mice appeared to be an excellent model to determine if MSH2 ATPase activity is required for CTG•CAG repeat expansions or if MSH2 binding to the trinucleotide repeats is sufficient to generate expansions in transgenic mice. We assessed intergenerational repeat instability over successive generations and somatic mosaicism in CTG repeat containing mice deficient for MSH2 ATPase activity. The *Msh2^G674A/G674A^* mice showed a dramatic decrease in CTG repeat expansions and an increase in contractions; a pattern paralleling that observed in *Msh2^−/−^* transgenic mice. Our data indicate that the ATPase-defective *Msh2^G674A^* mutation affects intergenerational and somatic instability of CTG•CAG repeats in transgenic mice.

## Results

**Figure 1:**
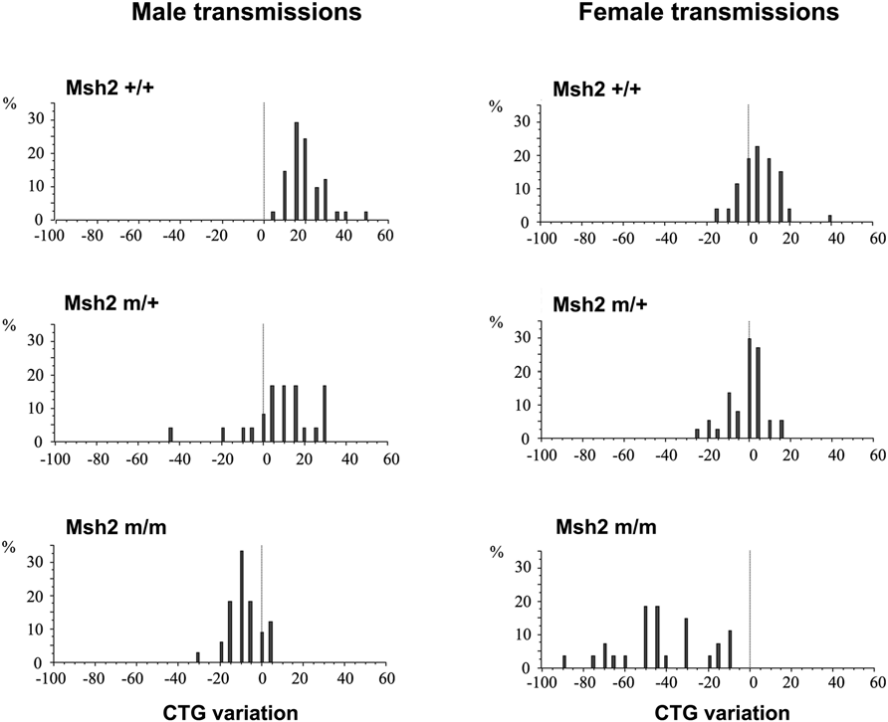
Intergenerational instability of CTG repeat in *Msh2^G674A^* mutant and wild-type mice. The x-axis shows CTG repeat length changes and the y-axis the percentage corresponding to each size changes for all transmissions analysed. Genotypes of parent and offspring are indicated on the left of each graph. For male transmissions, the transmitting progenitors were aged 14–52 weeks and carried an expanded trinucleotide sequence varying 395 to 563 CTG repeats. For female transmissions, the transmitting progenitors were aged 10–40.5 weeks and carried an expanded trinucleotide sequence varying from 401 to 647 CTG repeats.

To assess the consequence of the G674A *Msh2* missense mutation in the generation of expanded trinucleotide repeats, we crossed ATPase-defective *Msh2* mutant mice [27],[34] with DM300-328 mice carrying the human *DMPK* gene with >300 CTG repeats, which show high levels of intergenerational and somatic instability that is biased towards expansions and age-dependent [36]. We analyzed the length of the CTG repeat inherited from males and females, after weaning, in tail DNA from mice with different *Msh2* genotypes, as previously described [36]. The CTG instability in the various transmissions are reported in Table 1 and Figure 1. In *Msh2^+/+^* transgenic mice, we observed a high level of intergenerational instability biased towards expansions (100% and 62.3% expansions in offspring from male and female transmissions, respectively). In the *Msh2^G674A/+^* transmissions, the frequency of expansions significantly decreased for both male and female transmissions (from 100% to 75% and from 62.3% to 37.9%, respectively). For both paternal and maternal *Msh2^G674A/G674A^* transmissions, the frequency of expansions dramatically decreased (down to 12.1% and 0% in male and female transmissions, respectively). At the same time, the frequencies of CTG contractions increased in *Msh2^G674A/+^* and *Msh2^G674A/G674A^* transgenic mice. The distributions of the magnitudes of intergenerational CTG repeat length changes were significantly different between *Msh2* ^+/+^ and *Msh2^G674A/+^* and between *Msh2* ^+/+^ and *Msh2^G674A/G674A^* transmissions; *p*<0.005 and *p*<0.0001, respectively, both for male and female transmissions (assessed by the Mann-Whitney test). Interestingly, much larger contractions are observed in *Msh2^G674A/G674A^* female transmissions (with a mean of −41.9 CTG units). The sizes of contractions were positively correlated with the age of the transmitting female (r^2^ = 0.718, *p*<0.0001).

**Table 1:** Intergenerational CTG repeat instability in transgenic mice carrying the *G674A Msh2* mutation.

Parent to offspring transmission	Transmissions analysed (n)	Expansion frequency (%)	Contraction frequency (%)	Mean Expansion length (CTG)n	Mean Contraction length (CTG)n
**Male transmissions**					
*Msh2* +/+ *to* +/+	41	100	0	+19.5	
*Msh2 m/+ to m/+*	24	75	16.7	+15.6	−18.75
*Msh2 m/m to m/m*	33	12.1	78.8	+5	−11.2
**Female transmissions**					
*Msh2* +/+ *to* +/+	53	62.3	18.85	+10.8	−8
*Msh2 m/+ to m/+*	37	37.9	32.4	+7.1	−10.8
*Msh2 m/m to m/m*	27	0	100		−41.9

+/+/, m/+ and m/m represent specific genotype at the murine *Msh2* locus. +: wild-type allele; m: *G674A Msh2* mutation.

In summary, these data support the idea that the G674A mutation of one or both *Msh2* alleles significantly decreased the frequency of repeat expansions in paternal and maternal transmissions, suggesting that a functional MSH2 ATPase domain is required to generate expansions. Furthermore, these data indicate that the ATPase-mutant MSH2 is sufficient to lead to a preferential formation of CTG contractions that also arise in the *Msh2-null* and *Msh3-null* DM1 mice [19],[20]. Together these results support a requirement of a functional MSH2 ATPase domain for the expansion-biased parent to offspring CTG transmissions, and that this defect is sufficient to cause the formation of transmitted CTG contractions, which also arise in the absence of either MSH2 of MSH3 [19],[20].

**Figure 2:**
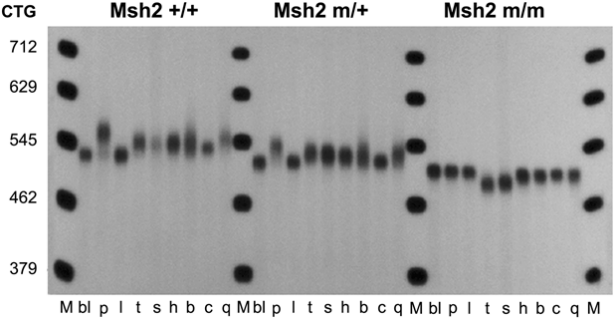
Somatic mosaicism in 8-month-old *Msh2^+/+^* ^,^ *Msh2^G674A/+^* and *Msh2^G674A/G674A^* transgenic males. bl, blood; p, pancreas; l, liver; t, testis; s, sperm; h, heart; b, brain; c, cerebellum; q, quadriceps. M. 250 bp DNA ladder converted to number of CTG repeat units in the given PCR product. *Msh2* +/+/, m/+ and m/m represent genotypes at the murine locus for *Msh2*. +: wild-type allele; m: *Msh2^G674A^* mutation. The Msh2 +/+, m/+ and m/m males in tail extracted DNA at weaning contained 525, 516 and 500 CTG repeats, respectively.

**Figure 3:**
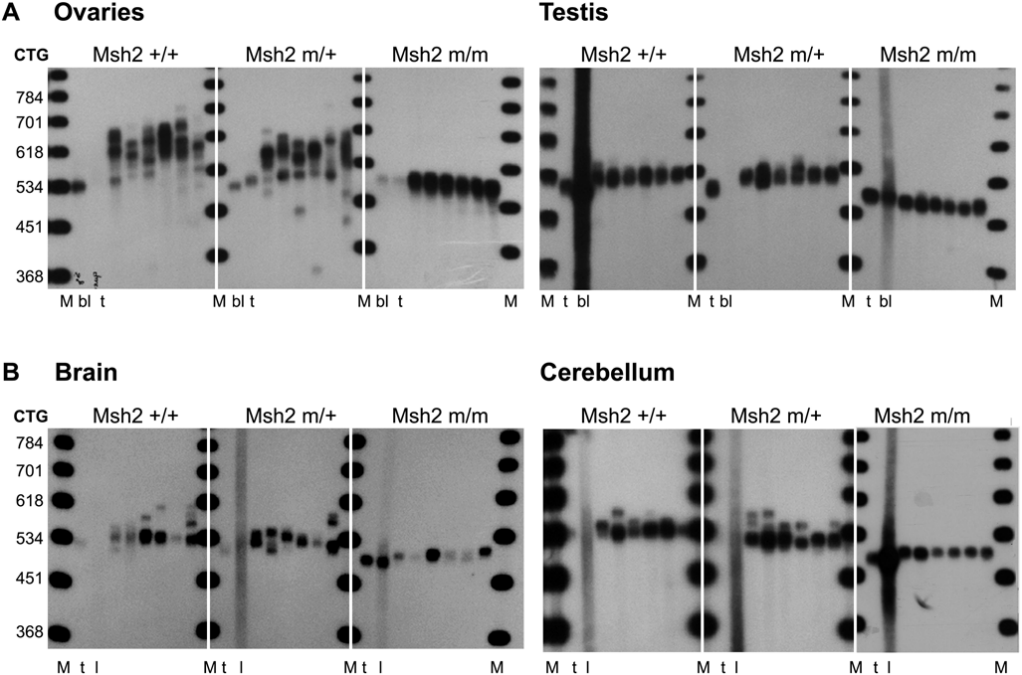
Representative SP-PCR analyses of CTG repeats in DNA molecules extracted from 8-month-old transgenic mouse tissues. The autoradiographs show representative SP-PCR analyses of DNA, extracted from ovaries and testis (A), brain and cerebellum (B). Genotypes are indicated. CTG repeat numbers determined in tail DNA at weaning are: 535 (*Msh2^+/+^);* 512 (*Msh2^m/+^*); 499 (*Msh2^m/m^*) in mice used to analyse DNA from ovaries and 525 (*Msh2^+/+^*); 516 (*Msh2^m/+^*); 500 (*Msh2^m/m^*) in the mice used for other tissues. t, tail; bl, blood; l, liver; M. 250 bp DNA ladder converted to number of repeat units in the given PCR product. About 2–10 amplifiable DNA molecules (ovaries, brain and cerebellum) and 10–50 transgene molecules (testis) were amplified. *Msh2+/+, m/+* and *m/m* represent genotypes at the murine locus for *Msh2*. +: wild-type allele; m: *Msh2^G674A^* mutation.

Both MSH2 and MSH3 proteins are required for CTG expansions in somatic tissues [19],[20],[37], yet the requirement of the MSH2 ATPase domain is unknown. Thus, we analysed the somatic CTG instability in several tissues collected from 8-month-old transgenic mice with different *Msh2* genotypes. Wild-type mice show inter- and intra-tissue CTG length mosaicism biased towards expansions, pronounced in pancreas and germinal tissues, as previously observed [19],[36] (Figure 2 and data not shown for female mice). A striking change in somatic instability was not readily observed by standard PCR for the *Msh2^G674A^* ^/+^ mice. In contrast, expansions were clearly not detected in *Msh2^G674A/G674A^* tissues and contractions were easily observed in testis and sperm, but not in somatic tissues. Somatic instability was assessed with greater sensitivity using small-pool PCR (SP-PCR). Tissues assessed by SP-PCR include ovaries, testis, brain and cerebellum; tissues known to display a range of instability levels in repair-proficient mice. The inherited size in each mouse is represented by the CTG repeat length in blood, liver or in tail at weaning, in which somatic instability is minimal throughout the animal's life [19]. In *Msh2^G674A/+^* mice, the degree of somatic mosaicism seems to be unchanged in all tissues analysed, and where present showed an expansion bias (Figure 3). In contrast, the degree of somatic CTG mosaicism was strikingly decreased in all tissues tested from the *Msh2^G674A/G674A^* mice. Neither expansions nor high levels of contractions were evident by either standard PCR or SP-PCR for the ATPase mutant mice, except in brain, where very small expansions could be detected. The absence of expansions in *Msh2^G674A/G674A^* mice parallels their loss in *Msh2^−/−^* mice. However, the absence of contractions in somatic tissues of *Msh2^G674A/G674A^* mice contrasts with their detection in tissues of *Msh2^−/−^* mice [19]. These results show that the homozygous *Msh2^G674A^* mutation reduces the formation of CTG•CAG expansions in somatic tissues, while mutation of a single allele has no significant effect. These results also indicate that the *Msh2^G674A/G674A^* mutation does not lead to high levels of CTG•CAG contractions in somatic tissues, which contrasts with their formation in tissues of *Msh2^−/−^* mice [19].

**Figure 4:**
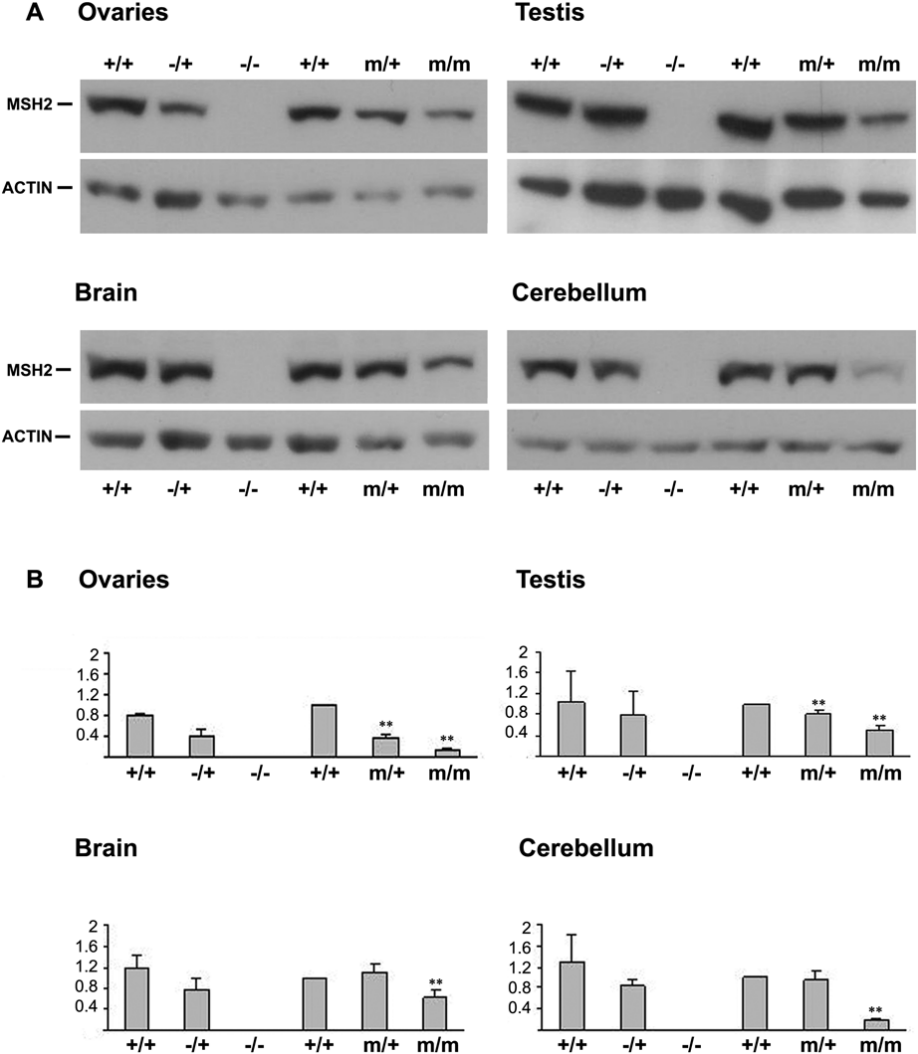
MSH2 protein levels in 3 to 4-month-old mice carrying different Msh2 genotypes. (A) Western blot analysis of MSH2 protein levels in ovaries, testis, brain and cerebellum from *Msh2^+/+^, Msh2^−/+^, Msh2^−/−^* (C57BL/6J, 129/OLA, FVB), *Msh2^+/+^, Msh2^m/+^* and *Msh2^m/m^* (>90% C57BL/6J) mice. β-actin was used as a loading control. Each lane represents pooled protein extracts from three mice. MSH2: 104 kDa and β-actin: ±42kDa. (B) The graphs show the quantitative analysis of MSH2 protein expression levels relative to β-actin in the tested tissues. The quantification has been performed by western blotting reproduced at least three times for each tissue. The x-axis shows MSH2/β-actin ratio. For each graph, all values have been calculated considering the value of *Msh2^+/+^* (C57BL/6J) as the reference value 1. **, *p*<0.005. *+/+, m/+* and *m/m* represent genotypes at the murine locus for *Msh2*. +: wild-type allele; m, *Msh2^G674A^* mutation. Error bars correspond to S.D.

**Figure 5:**
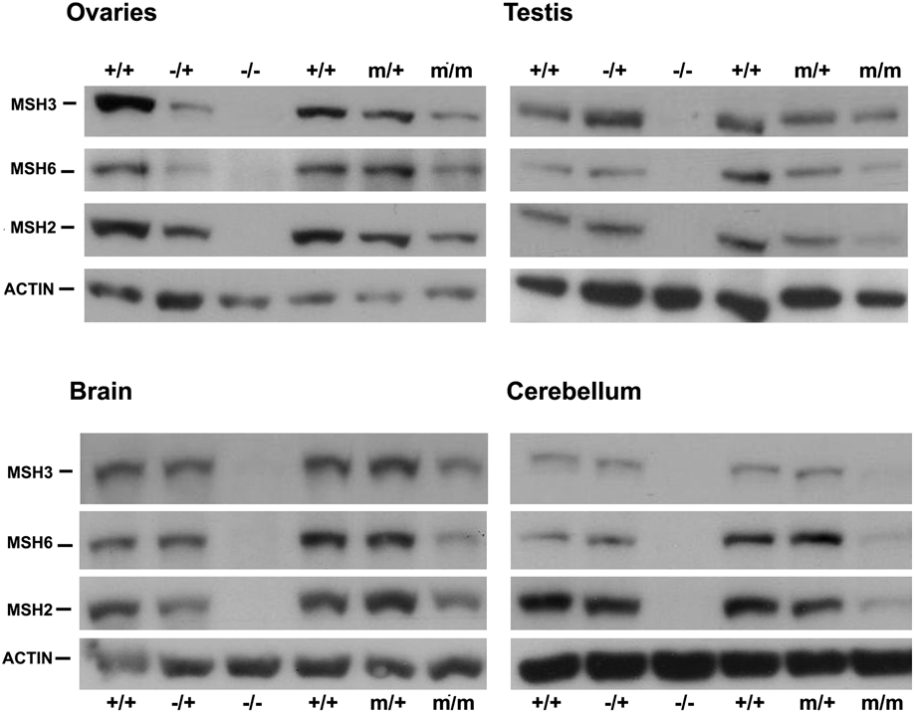
MSH2, MSH3 and MSH6 protein levels in 3 to 4-month-old mice carrying different *Msh2* genotypes. Western blot analysis of MSH2, MSH3 and MSH6 protein levels in ovaries, testis, brain and cerebellum from *Msh2^+/+^, Msh2^−/+^, Msh2^−/−^* (C57BL/6J, 129/OLA, FVB), *Msh2^+/+^, Msh2^m/+^* and *Msh2^m/m^* (>90% C57BL/6J) mice. β-actin was used as a loading control. Each lane represents pooled protein extracts from three mice. MSH2: 104 kDa, MSH3: 123 kDa, MSH6: 160kDa and β-actin: ±42kDa.

We have shown that CTG instability is dramatically decreased in *Msh2^G674A/G674A^,* supporting the idea that the G674A mutation in MSH2 ATPase domain affects CTG repeat instability. Lin *et al.* demonstrated that this mutant ATPase domain alters the MMR pathway in *Msh2^G674A/G674A^* mice without affecting the mismatch binding activity [34]. The stability of MSH2, MSH6 and MSH3 proteins depends on the presence of each partner involved in the formation of the MutSα and MutSβ complexes [38],[39]. In embryonic fibroblasts from *Msh2^G674A/G674A^* mice, expression levels of MSH2 and MSH6 proteins were normal, suggesting that the ATPase mutation did not alter the stability of these proteins [34]. We assessed the amount of MSH2 protein in germinal and cerebral tissues from 3 to 4-month-old transgenic mice with different *Msh2* genotypes, by western blot. We observed a decrease of MSH2 protein in *Msh2^G674A/G674A^* germinal tissues (to 13% in ovaries, to 50% in testis, *p*<0.001, *t*-test), cerebellum (to 19% *p*<0.001, *t*-test) and brain (to 62%, *p* = 0.005, *t*-test) (Figure 4). In mice heterozygous for the G674A mutation, MSH2 protein levels were also decreased only in germinal tissues (to 37% and 80% in ovaries and testis respectively, *p*<0.001, *t*-test). No detectable variation in protein levels was observed in brain and cerebellum of heterozygous mice. To ensure that the observed decrease of MSH2 protein was not due to a decreased sensitivity/avidity of the mutant MSH2 to the anti-MSH2 antibody, we also assessed the levels of MSH3 and MSH6 proteins and found these to be decreased in tissues of the *Msh2* ATPase-mutant mice with a pattern similar to the decrease of MSH2 (Figure 5).

To determine if the mutation decreases MSH2 expression at the RNA level, we measured transcript levels in *Msh2^G674A/G674A^* and wild-type tissues by qRT-PCR. Our analysis showed that the quantity of *Msh2^G674A^* transcripts is unchanged in homozygous mutant mice compared to wild-type mice (data not shown). Therefore, our results suggest that the missense mutation in MSH2 ATPase domain probably destabilizes MSH2 protein in adult mice.

## Discussion

Since the discovery of dynamic mutations, which are responsible for more than 20 neurological and/or neuromuscular diseases, many studies have focused on understanding the molecular mechanism of instability. Various data have clearly revealed that MSH2, MSH3, MSH6 and PMS2 are required for CTG•CAG expansions in mouse model systems [15]–[20]. However, the mode of action of these proteins in the generation of trinucleotide expansions remains unclear. It has been reported that MSH2-MSH3 ATPase activity was reduced upon binding to CAG hairpins, this was interpreted as impairing the repair of the CAG hairpin permitting its integration as an expansion [18]. In contrast, MutSβ was not required for either the correct or protected repair of slipped-CTG•CAG repeats, suggesting that MutSβ may be required to form these DNA structures with an unknown involvement of ATP hydrolysis [33]. Nevertheless, the role of the MutL homologue PMS2 in the formation of somatic expansions suggests that MMR pathway could be involved in the mechanism of instability [17]. In our study, we have shown that the G674A mutation in MSH2 ATPase domain strongly affects the formation of intergenerational expansions. *Msh2^G674A/G674A^* transgenic mice showed a strong bias towards contractions compared to *Msh2^+/+^* transgenic mice which showed a strong bias towards expansions in their offspring. In the homozygous *Msh2^G674A/G674A^* mice, we observed only 12.1% of expansions for paternal transmissions and no expansions for maternal transmissions (versus 100% and 62.3% in both male and female wild-type transmissions, respectively). These results suggested that a functional MSH2 ATPase domain is necessary to generate intergenerational expansions. These results also suggest that the defective ATPase domain is sufficient to yield a contraction bias similar to the *Msh2*-null mice. In testis, where 89.3% of seminiferous tubules are germinal cells [40], we observed a decrease of MSH2 levels of ∼50% in the *Msh2^G674A/G674A^* mice. This coupled with the severe reduction of expansions upon male *Msh2^G674A/G674A^* transmissions (from 100% for *Msh2^+/+^* down to 12% for the mutant mice) compared to the negligible effect for *Msh2^−/+^* mice (92%, Table S1 and [19]) strongly argue that a functional ATPase activity is required for expansions. Then, the deficiency of the ATPase domain in conjunction with decreased protein levels are responsible for the decrease of expansion frequency. Furthermore, it demonstrates that the apoptosis signaling function of MSH2, described to be non affected by the G674A mutation [34], is not involved in the mechanism generating CTG repeat expansions.

We also observed that the G674A mutation of one *Msh2* allele was sufficient to decrease the frequency of expansions in both male and female transmissions (from 100% to 75% expansions and from 62.3% to 37.9% for male and female transmissions, respectively). Such a decrease in the frequency of expansions was not observed for male transmissions in heterozygous *Msh2^−/+^* mice which displayed 92% expansions similar to *Msh2^+/+^* mice; indicating that in this context a single functional *Msh2* allele was sufficient to drive the same level of expansions as two functional alleles [19]. In contrast, our results show that a functional MSH2 protein in the presence of an ATPase mutant MSH2^G674A^ protein was not sufficient to drive the same levels of expansions; but yielded reduced expansion levels for paternal and maternal transmissions – further supporting a requirement of the ATPase domain. To this degree our data suggest that the MSH2^G674A^ mutant protein could act as a dominant-negative mutant, effectively blocking the ability of the wild-type MSH2 protein from driving CTG•CAG expansions. This purported dominant negative function would be specific to CTG•CAG expansions, as a dominant-negative mutator effect was not observed for either mono- or dinucleotide microsatellites in the heterozygous *Msh2^G674A/+^* mice [34]. In concordance with our observed CTG•CAG effect, the *S*. *cerevisiae* yMsh2 protein with the G693A (equivalent to the mammalian G674A mutation) or G693D mutated ATPase domains cause a dominant mutator phenotype [41],[42]. The mutant yMsh2^G693A^-yMsh6 complex binds to DNA mismatches and is not released in the presence of ATP. This could block access of wild-type proteins and subsequently inhibit normal repair [43]. In the case of CTG•CAG repeats, the MSH2^G674A^ mutant protein could partially block the access of the wild-type MSH2-MSH3 complex to unpaired CAG repeats, therefore protecting it from being processed to expansions. There is no data concerning the ability of the MSH2^G674A^-MSH3 complex to bind trinucleotide repeats. The analogous mutation in the conserved Walker domain of the prokaryote MutS protein (K620M) results in a weak mismatch binding activity and in a decrease in ATP binding and hydrolysis [24],[44]. However, in the case of MutS homoduplex in bacteria, both subunits of the complex are affected by the mutation. In eukaryotes, MSH2 partners (MSH3 or MSH6) retain their capacity to bind and hydrolyse ATP and it has been shown that MSH6 binds ATP with higher affinity and hydrolyses ATP faster than MSH2 [45]. Mispair binding is regulated by mismatch-stimulated ADP to ATP exchange inducing conformational changes to the N-terminus mispair binding domain [24],[46]. Recent data have shown that human MutSα and MutSβ have different ADP binding and ATPase activities *in vitro* that could explain the preferential processing of base-base and insertion/deletion mispairs by these two complexes [25]. In the mice used in this study, the MSH2^G674A^-MSH6 complex is able to bind to mismatches but the ATP-mediated disassociation from the substrate containing a G/T mismatch is reduced [34]. It is not known if the binding of MSH2^G674A^-MSH3 is affected by the mutation. However, the possible dominant-negative function of MSH2^G674A^ on CTG•CAG expansions suggest that MSH2^G674A^-MSH3 may still be able to bind to trinucleotide repeat expansions.

In *Msh2^G674A/G674A^* mice, we observed a dramatic decrease of somatic CTG expansions together with a decrease of MSH2 protein levels in adult somatic tissues, such as brain and cerebellum. Because the stability of MSH2, MSH3 and MSH6 depends on the ability of these proteins to form heterodimers, the levels of MSH3 and MSH6 were also found decreased in mutant tissues. However, MSH3 and MSH6 were clearly detected in homozygous mutant mice showing that the mutant MSH2^G674A^ protein was still able to form the MutSβ and MutSα complexes. While this reduction varied between tissues, a role of the ATPase domain in somatic instability seemed to be implicated. The dramatic loss of CTG instability in *Msh2^G674A/G674A^* mouse brains, which show as much as 62% of MSH2 protein strongly suggests that a functional ATPase domain is required for the formation of somatic expansions. The decrease of protein levels in the germinal and somatic tissues of *Msh2^G674A/G674A^* mice was unexpected as no decrease was observed in MEF cells from these mice [34]. Furthermore, Ollila *et al* recently observed that the production of human recombinant MSH2^G674A^-MSH6 complex was expressed in amount similar to the wild-type hMutSα suggesting that the G674A mutation did not affect protein stability [47]. However, a lowered interaction of MSH2^G674A^ with MSH6 was reported in this study. Interestingly, the difference in hMutSα expression levels between various cell lines was shown to result from cell-dependent differences in the degradation rate of both proteins forming the complex, by the ubiquitin-proteasome pathway [48]. Furthermore, the protein kinase PKCζ is able to phosphorylate hMutSα and regulates MSH2 and MSH6 protein stability and levels by inhibiting the ubiquitin-proteasome dependent degradation of these proteins [49]. It is unknown if the stability of MSH3 is regulated by the same pathway but the regulation of MSH2 stability has a direct impact on the regulation of MutSβ. It is possible that the *Msh2^G674A^* mutation affects the sensitivity of MSH2 to the ubiquitin-proteasome degradation pathways possibly by altering its ability to be phosphorylated. In this manner a tissue-specificity in the efficiency of the ubiquitin-proteasome degradation pathways would account for the different decrease of MSH2^G674A^ protein that we observed in the various mouse tissues. Interestingly, we found a putative PKC phosphorylation site at threonine 677; proximal to the G674A mutation, in the human and mouse MSH2 proteins using the NetPhosK 1.0 server (output score 0.89 using the Evolutionary Stable Sites filter) [50]. While tissue-specific regulation of MSH2 protein levels is beyond the scope of the present study, it is clear that the altered CTG instability we observe with the *Msh2^G674A^* mutation indicates that an ATPase-competent MSH2 protein is necessary to drive CTG•CAG expansions and that in its absence contractions predominate in parent-to-offspring transmissions.

It is noteworthy, that the levels of CTG•CAG contractions in either *Msh2^G674A/G674A^*, or *Msh3-null* mice is considerably greater in parent-offspring transmissions than in their somatic tissues ([20] and this study). The formation of high levels of contractions of expanded CTG•CAG repeats transmitted from parent-to-offspring depends upon the homozygous loss of *Msh2* or *Msh3*, or a homozygous *Msh2^ G674A/G674A^* mutation, and occur to some degree in a heterozygous *Msh2^G674A/+^* mutant. One interpretation is that the contractions would normally have been prevented by the presence of MSH3 and MSH2 with a functional ATPase domain. It would be of interest to learn if the germline-specific MSH2–MSH3:MLH3-MLH1/PMS2 complexes, detected at centromeric and Y chromosome repeats and proposed to prevent deleterious crossover events at repetitive DNAs [28], also form at expanded CTG•CAG transgenes in germ cells of transgenic mice. They may be linked to transmitted repeat contractions.

In conclusion, our finding that a functional MSH2 ATPase domain is required for CTG•CAG expansion mutations makes this the third mutagenic process for which its activity is required. The ATPase activity of MSH2 has been previously demonstrated to be required for both somatic hypermutation and class switch recombination at immunoglobin genes [26],[27]. Our data demonstrate that the *Msh2^G674A^* mutation affects CTG•CAG repeat instability in transgenic mice and almost completely abolishes the formation of intergenerational and somatic expansions and leads to a high level of contractions in parental transmissions. The G674A mutation also decreases the levels of MSH2 protein in a tissue-specific manner. The decrease of MSH2 protein cannot account totally for the dramatic decrease of expansions. Therefore, CTG•CAG repeat expansions are likely affected by cumulative effects of its defective ATPase domain and reduced MSH2 protein levels. If we assume that the binding of MSH2^G674A^-MSH3 is not affected by the mutation, our data would suggest that the binding of MutS complexes to the trinucleotide repeats is not sufficient to drive instability towards expansion and that a fully functional MSH2 ATPase domain is required. This does not support the hypothesis that a decrease of ATP hydrolysis by MSH2-MSH3 bound to trinucleotide repeats and aborted repair of DNA hairpin loops could cause expansions. On the contrary, our data sustain the hypothesis according to which a functional MMR activity is required to generate expansions.

## Materials and Methods

### Mouse Breeding and Genotyping

DM300-328 transgenic mice (>90% C57BL/6 background) carrying very large human genomic sequences (45 kb) and >300 CTG repeats [36] were crossed over successive generations with *Msh2^G674A^* mutant mice [27],[34]. Genotype was determined by PCR on 25 ng of DNA extracted from tail at weaning. Transgenic status of mice was performed with 0.5 µM of oligonucleotide primers HS3 (5′-TGAAGATGAAGCAGACGGG-3′) and HS4 (5′-TCCCCATTCACCAACAC-3′), 1× ReddyMix PCR Master Mix (Thermo Scientific). The DNA was denatured by heating to 94° for 6 min. Reactions involved 30 cycles of 94°C (30 s), 55°C (30 s) and 72°C (30 s) with a chase of 10 min at 72°C. *Msh2* genotype was determined with 0.35 mM of oligonucleotide primers in12/13 (5′-GTGGGTTTGTCTGACTGAATG-3′) and ex13/3 (5′ -GGATGGAAGAAGTCTCCAGC-3′), 1× ReddyMix PCR Master Mix and 2.5 mM of MgCl_2_. After initial denaturation for 10 min at 94°C, reactions were performed for 30 s at 94°C, 30 s at 60°C and 45 s at 72°C for 35 cycles followed by a chase of 7 min at 72°C. The sizes of predicted PCR products were 596 and 411 bp for mutant and wild-type alleles, respectively. *Msh2^−/+^* (129/OLA, FVB background) mutant mice and genotype by multiplex PCR have been described by de Wind *et al*. [51]. Housing and handling of mice were performed according to French government ethical guidelines.

### CTG Repeat Analysis

Intergenerational instability was investigated by comparison of the CTG repeat lengths in transgenic parent and offspring. DNA was extracted from tail collected at weaning by isopropanol precipitation. To determine the CTG repeat length in transgenic mice, 15 ng of tail DNA samples were amplified in 25 µl reaction using 0.4 µM 101 and 102 primers [36], 1× Custom master mix (Thermo Scientific) and 0.04U Thermoperfect *Taq* polymerase (Integro BV). The following cycling conditions were used: 5 min at 96°C; 45 s at 96°C, 30 s at 68°C and 3 min at 72°C (30 cycles); 1 min at 68°C and 10 min at 72°C (1 cycle). Electrophoresis of PCR products was performed using a previously described method [52]. The amplified product (from 2 µl to 5 µl) was mixed with DNA-loading dye and subjected to electrophoresis in 0.7–0.8% agarose gels at 300 V for 30 min and then 160 V overnight at 4°C. After electrophoresis, the gel was incubated in depurinating solution (0.25 M HCl) for 10 min, denaturing solution (0.5 M NaOH, 1.5 M NaCl) for 30 min and neutralization solution (1.5 M NaCl, 0.5 M Tris-HCl, pH 7.5) for 30 min. DNA was blotted onto positive nylon membrane (MP Biomedicals), UV-crosslinked (UV Stratalinker 2400, Stratagene) and hybridised with a double-strand CTG DNA probe radiolabelled with α-^32^P-dCTP using Amersham ready-to-go™ DNA labelling beads (-dCTG) (GE Healthcare). CTG repeat lengths were compared with a 250 bp DNA ladder loaded on the same gel and analysed by Quantity One-1-D Analysis Software (Bio-Rad Laboratories). Somatic instability was analysed in various tissues collected from 8-month-old mice. DNA was phenol/chloroform-extracted from tissues [14] and sperm DNA was extracted according to Seznec *et al*. [36]. CTG repeat instability was determined as described above.

### Small-Pool PCR (SP-PCR) Analysis

DNA extraction, SP-PCR amplifications and PCR product electrophoretic analyses were performed using the methods previously described [52]. DNA samples were digested with HindIII and SP-PCR was performed with DM-C and DM-BR primers [52]. SP-PCR products from mice with different *Msh2* genotypes and tail/blood or tail/liver PCR products were loaded on the same 0.7% agarose gel to compare instability between *Msh2^+/+^*, *Msh2^G674A/+^* and *Msh2^G674A/G674A^* mice.

### Protein Sample Preparation and Western Blotting

The different tissues were collected from 3 to 4-month-old-mice with different *Msh2* genotypes and pooled for protein extraction (three mice for each genotype). Proteins were extracted by mechanical homogenisation in lysis buffer (0.125 M Tris-HCl pH 6.8, 4% SDS, 10% glycerol) containing complete Mini 7× protease inhibitor cocktail (Roche) and 1 mM PMSF (Sigma). Protein concentration was determined using the Bio-Rad protein assay . 20 µg of proteins were denatured 5 min at 95°C in Laemmli sample buffer (Bio-Rad) supplemented with 5% β-mercaptoethanol added extemporaneously, resolved by electrophoresis on a 10% polyacrylamide SDS-PAGE gel and electroblotted onto Millipore Immobilon-P membranes (Millipore) in tranfer buffer (25 mM Tris-HCl pH 8.0, 192 mM glycine, 20% methanol and 0.1% SDS) at 350 mA at 4°C for 1h30. Membranes were blocked for one hour at room temperature in 5% blotto in TBST pH 7.5 ( 10 mM Tris-HCl, 0.15 mM NaCl and 0.05% Tween 20), then incubated overnight at 4°C in primary antibody. The membranes were washed once for 5 min, 10 min and 15 min each in TBST, incubated for 1h in secondary antibody (Jackson Immuno-research, sheep anti-mouse-HRP, 1∶5000) at room temperature for MSH2, MSH3, MSH6 and for actin, and washed one time for 5 min, 10 min and 15 min each in TBST. Antibody binding was visualized using ECL™ Western blotting analysis system and ECL plus Western blotting detection system (Amersham). MSH2, MSH6, and actin were detected using antibodies mouse anti-MSH2 (Oncogene, Ab-2, 1∶200), mouse anti-MSH6 (BD Laboratories, 1∶500), and mouse monoclonal β-actin (gift from Dr Manuel Hernàndez, 1∶400). The MSH3 monoclonal antibody was raised against an N-terminal fragment of mouse MSH3 (amino acids 24 to 308 of NCBI sequence NP_034959) and its specificity was confirmed using *Msh3* ^+/+^ and *Msh3* ^−/−^ mouse tissues (dilution 1∶500).

Western blotting was reproduced at least three times for each tissue to make semi-quantitative analysis. Densitometric analysis of protein levels was performed using Quantity One-1-D Analysis Software (Bio-Rad Laboratories) using non-saturated exposures.

### Statistical Analysis

Statistical analyses for instability distributions were performed with StatView software using the Mann-Whitney test (SAS Institute Inc.). The statistical analysis of the levels of MSH2 protein was performed with StatView software using *t*-test. The detected differences in total transmissions and in MSH2 protein levels were considered statistically significant only if *p*<0.05.

## Supporting Information

Table S1 Intergenerational CTG repeat instability in knockout *Msh2* mice.
(0.03 MB DOC)
