# Supplementary material for: MSH2 ATPase Domain Mutation Affects CTG•CAG Repeat Instability in Transgenic Mice
Source: PLoS Genet. 2009 May 15;5(5):e1000482. doi: 10.1371/journal.pgen.1000482 (PMC2674216; doi:10.1371/journal.pgen.1000482)
Supplement: Table S1 — Intergenerational CTG repeat instability in knockout Msh2 mice. (0.03 MB DOC) [file pgen.1000482.s001.doc]

| Supplementary Table I. Intergenerational CTG repeat instability in *Msh2* knockout mice | | | | | |
| --- | --- | --- | --- | --- | --- |
| **Parent to offspring transmission** | **Transmissions analysed**  **(n)** | **Expansion**  **frequency (%)** | **Contraction frequency (%)** | **Mean**  **Expansion length (CTG)n** | **Mean**  **Contraction length (CTG)n** |
| Male transmissions |  |  |  |  |  |
| *Msh2 -/+ to -/+* | 39 | 92 | 7 | +15.5 | -8 |
| *Msh2 -/- to -/-* | 51 | 2 | 92 | +2 | -12 |
| **Female transmissions** |  |  |  |  |  |
| *Msh2 -/+ to -/+* | 45 | 62 | 33 | +8 | -11 |
| *Msh2 -/- to -/-* | 31 | 2 | 90 | 2 | -18 |

-/+ and -/- represent specific genotype at the murine *Msh2* locus. +: wild-type allele;

-: *Msh2* knockout mutation.
